# Draft Genome Sequence of an Extended-Spectrum β-Lactamase-Producing Klebsiella oxytoca Strain Bearing *mcr-9* from Qatar

**DOI:** 10.1128/MRA.00429-20

**Published:** 2020-06-04

**Authors:** Clement K. M. Tsui, Sathyavathi Sundararaju, Hassan Al Mana, Mohammad Rubayet Hasan, Patrick Tang, Andres Perez-Lopez

**Affiliations:** a Department of Pathology, Sidra Medicine, Doha, Qatar; b Department of Pathology and Laboratory Medicine, Weill Cornell Medicine-Qatar, Doha, Qatar; c Division of Infectious Diseases, Faculty of Medicine, University of British Columbia, Vancouver, BC, Canada; d Biomedical Research Center, Qatar University, Doha, Qatar; Loyola University Chicago

## Abstract

Klebsiella oxytoca is an opportunistic human pathogen causing nosocomial infection. We report the draft genome of an extended-spectrum β-lactamase-producing K. oxytoca isolate harboring an *mcr-9* gene, a recently discovered colistin resistance analog, from Qatar. The genome statistics, along with the sequence type and resistance mechanisms, are predicted for the assembled genome.

## ANNOUNCEMENT

Colistin in combination with other agents is used for the treatment of severe infections caused by carbapenem-resistant Gram-negative bacteria. The emergence of plasmid-mediated resistance to colistin due to lipid A-modifying enzymes encoded by 10 different *mcr* genes in *Enterobacterales* has endangered the last-resort treatment (1–3). One of the genes, *mcr-9*, has been recently described in Escherichia coli, Klebsiella pneumoniae, *Enterobacter* spp., and Salmonella enterica serotype Typhimurium (4–9) from animals and humans. The increasing reports of novel *mcr* genes have become a great concern in many countries. Therefore, it is crucial to understand the molecular mechanisms of colistin resistance in Qatar.

Klebsiella oxytoca is an opportunistic pathogen associated with nosocomial infections (10, 11). It becomes a public health concern because of resistance to antimicrobials due to the presence of *bla*_OXY_, *bla*_CTX-M_, and *bla*_SHV_ genes (11). Here, we report the genome of K. oxytoca strain 18099069b harboring *mcr-9*, which was isolated from a rectal swab obtained from a child during admission to the intensive care unit to screen for extended-spectrum β-lactamase (ESBL)- and carbapenemase-producing *Enterobacteriaceae*. Swabs are routinely inoculated onto CHROMagar ESBL and CHROMagar mSuperCARBA (CHROMagar, France). Plates are incubated under aerobic conditions at 35 ± 2°C for 18 to 24 h, minimizing exposure to light. Suspicious colonies growing on the chromogenic agar plates after overnight incubation are identified using the matrix-assisted laser desorption ionization–time of flight (MALDI-TOF) Biotyper system (Bruker, Bremen, Germany). In this case, MALDI-TOF mass spectrometric identification was performed on a single colony picked from the ESBL chromogenic plate onto the MALDI target plate. Antimicrobial susceptibility testing was performed using the Phoenix system (Becton, Dickinson, USA). The MIC for colistin was determined by broth microdilution (ComASP Colistin; Liofilchem, Italy). MICs were interpreted according to the CLSI breakpoints (12). Ethics approval for the study was obtained from the institutional review board of Sidra Medicine.

DNA was extracted using the NucliSENS easyMag platform (bioMérieux, France). DNA libraries were constructed with a Nextera XT kit (Illumina, CA) and sequenced on the Illumina MiSeq system with 2 × 300 cycles. The sequence data were trimmed using Trim Galore v0.6.1 (http://www.bioinformatics.babraham.ac.uk/projects/trim_galore), assembled using SPAdes v3.9.0 (13), and assessed using QUAST v5.0.2 (14). Contaminant data were analyzed by Kraken v2 (15). The sequence type (ST), plasmids, and antimicrobial resistance (AMR) genes were predicted using the mlst (https://github.com/tseemann/mlst), PlasmidFinder v2.1 (16), and ResFinder v3.2 (17) databases in abricate v0.9.8 (https://github.com/tseemann/abricate), based on ≥60% coverage and 90% sequence identity. The repeat sequences of strains were identified using ISfinder v2 (18). The assembled genome was annotated by the NCBI Prokaryotic Genome Annotation Pipeline v4.11 (19). Default parameters were used for all software unless otherwise specified.

The genome statistics for 18099069b are summarized in Table 1. This isolate contained *bla*_TEM1B_, *bla*_SHV12_, and *bla*_OXY22_, encoding different class A β-lactamases, which confer resistance to not only narrow-spectrum β-lactams but also broad-spectrum cephalosporins (Table 1). This strain was susceptible to colistin with a MIC of 2 μg/ml, consistent with the report that resistance to colistin (MIC, ≥4 μg/ml) in the presence of *mcr-9* occurs only when the regulatory genes *qseC* and *qseB* are expressed (5). A BLAST search of the contig (2,659 bp) containing *mcr-9* indicated 100% identity to plasmids of multiple bacteria; two of them (GenBank accession numbers CP043767.1 and CP044215.1) were annotated as replicon type IncHI2, which is well known for its association with *bla*_SHV-12_ and *mcr-9* (6). Insertional elements IS*903* and IS*15* were also detected in the flanking regions of *mcr-9*.

**TABLE 1:** Summary of genome statistics and genetic mechanism of antibiotic resistance

Parameter	Observation
Isolate	18099069b
No. of reads	1,526,962
Genome size (bp)	6,190,059
*N*_50_ (bp)	210,467
No. of contigs	95
Avg coverage (×)	135
GC content (%)	54.8
No. of coding sequences	5,792
ST	58
Plasmid types	IncHI2, IncHI2A, IncFIB(K)
AMR genes	*bla*_OXY2-2_, *bla*_SHV-12_, *bla*_TEM-1B_, *aac(6')-IIc*, *aph(3')-Ia*, *aac(3)-IIb*, *ere*(A), *oqxAB*, *fosA*, *sul1*, *sul2*, *catA2*
Antibiotic resistance phenotypes^*a*^	AMP, CAZ, PIP, GEN, SXT

a AMP, ampicillin; CAZ, ceftazidime; PIP, piperacillin; GEN, gentamicin; SXT, trimethoprim-sulfamethoxazole.

### Data availability.

This whole-genome shotgun project has been deposited in DDBJ/ENA/GenBank under the accession number JAAWXG000000000, as well as accession numbers PRJNA577539 and SRR11485633. The version described in this paper is version JAAWXG010000000.

## References

[1] Liu Y-Y, Wang Y, Walsh TR, Yi L-X, Zhang R, Spencer J, Doi Y, Tian G, Dong B, Huang X, Yu L-F, Gu D, Ren H, Chen X, Lv L, He D, Zhou H, Liang Z, Liu J-H, Shen J 2016 Emergence of plasmid-mediated colistin resistance mechanism *MCR-1* in animals and human beings in China: a microbiological and molecular biological study. Lancet Infect Dis 16:161–168. doi:10.1016/S1473-3099(15)00424-7. 26603172

[2] Sun J, Zhang H, Liu Y-H, Feng Y 2018 Towards understanding MCR-like colistin resistance. Trends Microbiol 26:794–808. doi:10.1016/j.tim.2018.02.006. 29525421

[3] Wang C, Feng Y, Liu L, Wei L, Kang M, Zong Z 2020 Identification of novel mobile colistin resistance gene *mcr-10*. Emerg Microbes Infect 9:508–516. doi:10.1080/22221751.2020.1732231. 32116151 PMC7067168

[4] Wang Y, Liu F, Hu Y, Zhang G, Zhu B, Gao GF 2020 Detection of mobile colistin resistance gene *mcr-9* in carbapenem-resistant *Klebsiella* pneumoniae strains of human origin in Europe. J Infect 80:578–606. doi:10.1016/j.jinf.2019.12.016. 31891728

[5] Faccone D, Martino F, Albornoz E, Gomez S, Corso A, Petroni A 2020 Plasmid carrying *mcr-9* from an extensively drug-resistant NDM-1-producing *Klebsiella quasipneumoniae* subsp. *quasipneumoniae* clinical isolate. Infect Genet Evol 81:104273. doi:10.1016/j.meegid.2020.104273. 32145334

[6] Chavda KD, Westblade LF, Satlin MJ, Hemmert AC, Castanheira M, Jenkins SG, Chen L, Kreiswirth BN 2019 First report of *bla*_VIM-4_- and *mcr-9*-coharboring *Enterobacter* species isolated from a pediatric patient. mSphere 4:e00629-19. doi:10.1128/mSphere.00629-19. 31511372 PMC6739498

[7] Yuan Y, Li Y, Wang G, Li C, Xiang L, She J, Yang Y, Zhong F, Zhang L 2019 Coproduction of MCR-9 and NDM-1 by colistin-resistant *Enterobacter hormaechei* isolated from bloodstream infection. Infect Drug Resist 12:2979–2985. doi:10.2147/IDR.S217168. 31571950 PMC6756836

[8] Carroll LM, Gaballa A, Guldimann C, Sullivan G, Henderson LO, Wiedmann M 2019 Identification of novel mobilized colistin resistance gene *mcr-9* in a multidrug-resistant, colistin-susceptible *Salmonella enterica* serotype Typhimurium isolate. mBio 10:e00853-19. doi:10.1128/mBio.00853-19. 31064835 PMC6509194

[9] Kieffer N, Royer G, Decousser J-W, Bourrel A-S, Palmieri M, Ortiz De La Rosa J-M, Jacquier H, Denamur E, Nordmann P, Poirel L 2019 *mcr-9*, an inducible gene encoding an acquired phosphoethanolamine transferase in *Escherichia coli*, and its origin. Antimicrob Agents Chemother 63:e00965-19. doi:10.1128/AAC.00965-19. 31209009 PMC6709461

[10] Högenauer C, Langner C, Beubler E, Lippe IT, Schicho R, Gorkiewicz G, Krause R, Gerstgrasser N, Krejs GJ, Hinterleitner TA 2006 *Klebsiella oxytoca* as a causative organism of antibiotic-associated hemorrhagic colitis. N Engl J Med 355:2418–2426. doi:10.1056/NEJMoa054765. 17151365

[11] Izdebski R, Fiett J, Urbanowicz P, Baraniak A, Derde LPG, Bonten MJM, Carmeli Y, Goossens H, Hryniewicz W, Brun-Buisson C, Brisse S, Gniadkowski M 2015 Phylogenetic lineages, clones and β-lactamases in an international collection of *Klebsiella oxytoca* isolates non-susceptible to expanded-spectrum cephalosporins. J Antimicrob Chemother 70:3230–3237. doi:10.1093/jac/dkv273. 26318191

[12] Clinical and Laboratory Standards Institute. 2018 Performance standards for antimicrobial susceptibility testing: 28th informational supplement. M100-S23. Clinical and Laboratory Standards Institute, Wayne, PA.

[13] Bankevich A, Nurk S, Antipov D, Gurevich AA, Dvorkin M, Kulikov AS, Lesin VM, Nikolenko SI, Pham S, Prjibelski AD, Pyshkin AV, Sirotkin AV, Vyahhi N, Tesler G, Alekseyev MA, Pevzner PA 2012 SPAdes: a new genome assembly algorithm and its applications to single-cell sequencing. J Comput Biol 19:455–477. doi:10.1089/cmb.2012.0021. 22506599 PMC3342519

[14] Gurevich A, Saveliev V, Vyahhi N, Tesler G 2013 QUAST: quality assessment tool for genome assemblies. Bioinformatics 29:1072–1075. doi:10.1093/bioinformatics/btt086. 23422339 PMC3624806

[15] Wood DE, Salzberg SL 2014 Kraken: ultrafast metagenomic sequence classification using exact alignments. Genome Biol 15:R46. doi:10.1186/gb-2014-15-3-r46. 24580807 PMC4053813

[16] Carattoli A, Zankari E, García-Fernández A, Voldby Larsen M, Lund O, Villa L, Møller Aarestrup F, Hasman H 2014 In silico detection and typing of plasmids using PlasmidFinder and plasmid multilocus sequence typing. Antimicrob Agents Chemother 58:3895–3903. doi:10.1128/AAC.02412-14. 24777092 PMC4068535

[17] Zankari E, Hasman H, Cosentino S, Vestergaard M, Rasmussen S, Lund O, Aarestrup FM, Larsen MV 2012 Identification of acquired antimicrobial resistance genes. J Antimicrob Chemother 67:2640–2644. doi:10.1093/jac/dks261. 22782487 PMC3468078

[18] Siguier P, Perochon J, Lestrade L, Mahillon J, Chandler M 2006 ISfinder: the reference centre for bacterial insertion sequences. Nucleic Acids Res 34(Suppl 1):D32–D36. doi:10.1093/nar/gkj014. 16381877 PMC1347377

[19] Tatusova T, DiCuccio M, Badretdin A, Chetvernin V, Nawrocki EP, Zaslavsky L, Lomsadze A, Pruitt KD, Borodovsky M, Ostell J 2016 NCBI Prokaryotic Genome Annotation Pipeline. Nucleic Acids Res 44:6614–6624. doi:10.1093/nar/gkw569. 27342282 PMC5001611

